# Mechanisms and Therapeutic Targets of Depression After Intracerebral Hemorrhage

**DOI:** 10.3389/fpsyt.2018.00682

**Published:** 2018-12-17

**Authors:** Yinan Wu, Liangliang Wang, Kaimin Hu, Chengcheng Yu, Yuanhan Zhu, Suzhan Zhang, Anwen Shao

**Affiliations:** ^1^Cancer Institute, Key Laboratory of Cancer Prevention and Intervention, China National Ministry of Education, The Second Affiliated Hospital, School of Medicine, Zhejiang University, Hangzhou, China; ^2^Interdisciplinary Institute of Neuroscience and Technology, Qiushi Academy for Advanced Studies, Zhejiang University, Hangzhou, China; ^3^Department of Surgical Oncology, The Second Affiliated Hospital, School of Medicine, Zhejiang University, Hangzhou, China; ^4^Department of Orthopedics, The Second Affiliated Hospital, School of Medicine, Zhejiang University, Hangzhou, China; ^5^Department of Neurosurgery, Rongjun Hospital, Jiaxing, China; ^6^Department of Neurosurgery, The Second Affiliated Hospital, School of Medicine, Zhejiang University, Hangzhou, China

**Keywords:** intracerebral hemorrhage, depression, inflammation, oxidative stress, apoptosis, review, therapeutic target, autophagy

## Abstract

The relationship between depression and intracerebral hemorrhage (ICH) is complicated. One of the most common neuropsychiatric comorbidities of hemorrhagic stroke is Post-ICH depression. Depression, as a neuropsychiatric symptom, also negatively impacts the outcome of ICH by enhancing morbidity, disability, and mortality. However, the ICH outcome can be improved by antidepressants such as the frequently-used selective serotonin reuptake inhibitors. This review therefore presents the mechanisms of post-ICH depression, we grouped the mechanisms according to inflammation, oxidative stress (OS), apoptosis and autophagy, and explained them through their several associated signaling pathways. Inflammation is mainly related to Toll-like receptors (TLRs), the NF-kB mediated signal pathway, the PPAR-γ-dependent pathway, as well as other signaling pathways. OS is associated to nuclear factor erythroid-2 related factor 2 (Nrf2), the PI3K/Akt pathway and the MAPK/P38 pathway. Moreover, autophagy is associated with the mTOR signaling cascade and the NF-kB mediated signal pathway, while apoptosis is correlated with the death receptor-mediated apoptosis pathway, mitochondrial apoptosis pathway, caspase-independent pathways and others. Furthermore, we found that neuroinflammation, oxidative stress, autophagy, and apoptosis experience interactions with one another. Additionally, it may provide several potential therapeutic targets for patients that might suffer from depression after ICH.

## Introduction

Each year, about 795,000 individuals suffer from a new or recurrent stroke. Nearly 610,000 among these patients experience first time attacks in their entire life; the remaining cases are reported as recurrent strokes. All stroke cases, 87% are ischemic, while intracerebral hemorrhage (ICH) strokes account for 10%, and subarachnoid hemorrhage (SAH) strokes only make up 3% of the total (1).

Post-stroke depression (PSD), the most common and frequent mental disorder after stroke, has a strong association with exacerbate deterioration of functional recovery, physical, and cognitive outcome, as well as quality of life. Moreover, PSD is even independently associated with enhanced morbidity, disability, and mortality (2–4).

Intracerebral hemorrhage (ICH) is a dangerous type of stroke, which is severer. Evidence have shown that 20% of ICH survivors existed with explicit signs of depression (5, 6). Numerous studies of PSD have been revealed, especially ischemic strokes; studies were based on medical examinations in researches. Nevertheless, the etiological factors that cause post-ICH depression are far from being elucidated. Hence, it is vital to understand the specific etiopathology of depression after ICH that can thus help people to develop effective therapeutic strategies aimed at etiological factors.

The present review will address the mechanisms, especially involved signaling pathways, and will introduce several potential therapeutic agents for the therapy of post-ICH depression. Finally, we will provide suggestions, that we hope can guide future research.

## ICH Types

ICH is divided into primary and secondary types, depending on the response to the fundamental cause of hemorrhage (7). Primary ICH develops without any underlying vascular malformation or coagulopathy. However, some cases like tumors, trauma, as well as coagulation could lead to the formation of secondary ICH, as well as the use of thrombolytic agents (7). In any ICH case, primary brain damage will occur because of the hemorrhage. And with the development of a hematoma, secondary brain injury will gradually appear on account of a pathological and physiological reaction.

Intracerebral hemorrhage is a lethal type of stroke, in the United States, every year there are 30,000 people who die from a stroke. If the patient is lucky enough to survive, then the growth of the hematoma in the brain parenchyma could trigger multiple of reactions that lead to another insult or even more severe neurological impairments. We will discuss several aspects of secondary cerebral injury following ICH and underline the key mechanisms correlated with post ICH depression (8).

## Secondary Brain Injury-Induced Depression

Secondary injury is a key factor in the deterioration of the nervous system in ICH patients (9, 10). Secondary brain injury after ICH is caused by intraparenchymal hemorrhage, which then activates several signaling pathways such as inflammatory, oxidative, autophagic, and apoptotic pathways. These pathways, *in vivo*, become the bridge that links intracerebral hemorrhage and depression (8, 11–13).

### Inflammation

Inflammation is a significant host defense response to cerebral damage following ICH. Once ICH occurs, components in the blood such as leukocytes, RBCs, and macrophages immediately migrate into the brain parenchyma. There is growing evidence that inflammation-induced impairment plays a crucial role in the mechanism underlying secondary brain injury after ICH (8, 14–17).

#### Toll-Like Receptors in Inflammation

Toll-like receptors (TLRs) are an important component of inflammatory responses and innate immunity (18, 19). TLR4 on leukocytes are important for the infiltration of both neutrophils and monocytes out of circulation (20). Recently, several clinical studies have suggested that increased levels of TLR2 and TLR4 expression in peripheral monocytes is related to a poor prognosis in patients with ICH (21). Furthermore, some studies have found an improved neurological function in TLR4-knockout ICH animal models (20, 22). Moreover, TLR4 signaling, especially those on resident microglia and on blood-derived inflammatory cells, is specific to inflammatory damage induced by ICH (20, 22, 23).

Recently, more attention has been placed on understanding the underlying mechanisms of inflammation-induced depression. Kéri et al. found patients diagnosed as major depressive disorder (MDD) for the first time, usually had an accompanied upregulation of TLR-4 signaling. It is thought to be related to bacterial translocation or various molecular patterns that correlate with the type of injury (24). Strekalova et al. first showed that C57BL/6J mice models appeared to show depression- and anxiety-like behaviors when they were fed high amounts of cholesterol. Moreover, they reported an unexpected elevation in the level of TLR4 expression, which indicated that TLR4 may play a critical role in the central neuronal system (25). Habib and his colleagues clarified, in an experiment using diabetic/depressed rats, when dysfunctions occurred to blood vessels as well as the metabolic system, the expression of TLR-4 in the aorta increased rapidly, in addition to a rise in pro-inflammatory cytokines (26). Later, Cheng et al. found that stress-induced neuroinflammatory responses are regulated by the GSK3-dependent TLR4 pathway. This signaling pathway is involved in development of depressive-like symptoms (27). García et al. then concluded that the activation of TLR-4 in the brain and peripheral area leads to sickness symptoms, and its expression level is also a risk factor that contributes to depression (28). These results confirmed the correlation between an elevated level of the TLRs and the risk of developing depression.

An increasing body of evidence suggests that microglia are the main mediators of inflammation-related disorders, including depression. Slusarczyk et al. suggested that tianeptine, an antidepressant drug, could attenuate the level of inflammatory mediators related to TLR4 signaling and the NLRP3 inflammasome (29). In addition, chronic restraint stress (CRS)-induced depressive-like animal models were found to show inflammatory responses in the hippocampus via the toll-like receptor type 4 (TLR4)/p38 mitogen-activated protein kinase (MAPK) pathway, which could be treated by ketamine (30). Past studies demonstrated that the TLR4 signaling pathway in the CNS and the periphery are associated with activated glycogen synthase kinase-3 (GSK3), a kinase shown to be involved in depression (31, 32). GSK3 inhibition has been indicated to reduce the production of pro-inflammatory cytokines with the stimulation of TLR4 in several different immune cells, both in clinical and basic experiments (33–35). Moreover, antidepressants like fluoxetine or the GSK3 inhibitor, TDZD-8, could improve stress-induced depressive-like behaviors via the TLR4 signaling pathway (27).

#### NF-kB Mediated Signal Pathway

Recently, many studies have proven the instrumental role of proinflammatory cytokines in the development of ICH. For instance, the activation of NF-kB in microglia/macrophages, which contributes to brain damage after ICH, results in the upregulation of proinflammatory cytokines (36, 37). Moreover, inhibited NF-kB activity is also related to alleviated neurological deficits (22, 38).

Furthermore, plenty of research suggests that neuroinflammation may play a significant role in the pathogenesis of depressive disorders. Koo et al. first reported that NF-κB signaling may act as a key mediator in anti-neurogenic and stress-induced behavioral actions; it may provide therapeutic targets of depression, which have never been described before (39). A few years after, evidence was provided that MDD is characterized by up-regulation of redox-sensitive transcriptional factors (Nrf2 and NF-κB), which indicated the pro-oxidative state that exists in MDD patients' peripheral blood mononuclear cells (PBMC) (40). A review concerned with adult hippocampal neurogenesis similarly supported the finding that NF-κB signaling modulates neurogenesis in adult patients, as well as expressing antidepressant actions (41). Recently, Nadeem et al. discovered that IL-17A seems to participate in comorbid depression with those who have psoriatic inflammation; this was linked to NF-κB and p38MAPK pathways that function through the up-regulated inflammatory cytokines in the brain (42). What is more, chronic stress in the basolateral amygdala (BLA) would induce the upregulation of neuropeptides and subsequently cause depressive-like behaviors. The siRNA could mediate the inhibition of NF-kB signaling in the BLA and downregulate the expression of neuropeptides, which lead to the alleviation of depressive symptoms (43). Moreover, Su et al. (44) proved that chronic unpredictable mild stress (CUMS)—induced depression-like action could be mediated through the NLRP3 inflammasome. Furthermore, the depression rat model indicated that the CUMS-induced MAPK pathway could be regulated by NLRP3 inflammasome by activating the NF-κB protein complex (44). Depression is one of the upmost psychological illness that is closely tied with inflammation. Crocin could act as a promising therapeutic target for depressive-like behaviors and neuro-inflammation caused by lipopolysaccharide (LPS). Researchers found such a phenomenon was an outcome of inhibited NLRP3 inflammasomes as well as inhibited NF-κB signaling in microglia (45). Pro-anthocyanidin, having potential anti-inflammatory and antioxidative activity efficacy, functions as an effective therapeutic candidate for depression-like behaviors induced by LPS by regulating the NF-κB signal in many cerebral regions and inhibiting the LPS-induced iNOS and the increased expression of COX-2 (46). Senegenin (SEN) is a main bioactive component of Polygala tenuifolia Willd, which has sturdy effects including anti-inflammatory actions as well as neuroprotection. At the same time, it has been used to lessen the depressive behavior in CUMS-induced rat models by inhibiting NLRP3-regulated NF-κB signaling (47). Icariin (ICA), which could be extracted from a certain traditional Chinese herb, is able to freely transverse the blood-brain barrier. It reduces neuroinflammation and OS-induced brain damage to prevent depressive-like behaviors by inhibiting the activation of NF-κB signaling in addition partially inhibition of the NLRP3-inflammasome/caspase-1/IL-1β axis, which would increase the antioxidant and anti-inflammatory ability of the cerebrum (48). With associated neuroprotection and anti-inflammatory activities, Geraniol (GE) has the potential to treat antidepressant-like behaviors in CUMS-induced depression mouse models, possibly by inhibiting the NF-κB pathway activation. Likewise, it seems that the regulation of nucleotide binding and NLRP3 inflammasome expression are both involved in this process (49). On the other hand, Chrysophanol (Chr) was also reported to function as anti-depression treatment by influencing the P2X7/NF-κB signaling pathway (50).

#### PPAR-γ-Dependent Pathway

CD36, belonging to the class B scavenger receptor family, is usually expressed in macrophages or microglia. It is involved in phagocytosis of many pathogens such as bacteria, apoptotic cells and oxidized low-density lipoproteins (51–53). Peroxisome proliferator-activated receptor (PPAR) -γ, which is a part of the nuclear hormone receptor superfamily, can transcriptionally regulate the expression of CD36 and participate in inflammation (54, 55). In addition, treated with PPARγ activator, the hematoma in the brain of ICH mouse model would regress quicker and neurological damage following ICH in adults would decline. Flores and his colleagues confirmed that PPARγ agonists (15d-PGJ2) raised short-term PPARγ levels, accompanied with enhanced CD36 expression and accelerated hematoma resolution. Furthermore, it improved neurological function results. Moreover, both long term ventricular dilatation after ICH and white matter loss were decreased (56).

In several clinical and basic experiments, PPAR-γ agonists have exerted anti-depressive behavioral effects. Nevertheless, no one explained these mechanisms clearly. Gold and his colleagues proposed that PPAR-γ may exhibit as a conceptually new remedial target that improves the affective, cognitive and systemic manifestations of MDD (57). Later, Agudelo et al. opened a novel therapeutic avenue for treating depression through the PGC-1α1-PPAR axis, which was usually expressed in skeletal muscles, rather than by crossing the blood-brain barrier (58). Colle et al. found that PPAR-γ agonists have antidepressant effects in 3 out of 4 RCTs and in 4 open-label studies. Consequent studies concluded that PPAR-γ agonists may have antidepressant effects (59). Recently, several studies suggested that PPAR-γ agonist exhibit their antidepressant-like effects through various pathways: Liao and his colleagues firstly revealed the regulation of the CREB/BDNF and NF-κB/IL-6/STAT3 pathways, together with the potential effects on central 5-HT neurotransmission may be implicated in depressive-like behaviors via PPAR-γ-related effects (60). Through the upregulation of PPARγ expression, Song and his colleagues proposed that neuroinflammation could be inhibited and even play a role in its antidepressant effects (61).

Selective agonists of the nuclear transcription factor PPAR-γ are used to treat type 2 diabetes. Several studies also seem to suggest their contribution to improvement of depressive symptoms. PPAR-γ agonist pioglitazone (60, 62, 63), rosiglitazone (64–66), Troglitazone (67), atorvastatin (68), folic acid (69), Astragaloside IV (61), all of which have been reported to ameliorate depressive-like behaviors in mice via the PPAR-γ inflammasome axis.

#### Other Pathways

Inhibiting transient receptor potential Classic 3 (TRPC3), a member of the calcium-permeable cation channels, significantly reduced the perihematomal accumulation of reactive astrocytes, indicating that TRPC3 plays an important part in activating astrocytes following ICH. Accumulating findings indicate that neurological functions improve with reduced cerebral edema by inhibiting activated astrocytes via the TRPC3 inhibitor Pyr3.

In recent years, several studies have reported that the alterations of intracellular Ca^2+^ signaling are the basis for the pathophysiology of psychiatric disorders, including depression (70, 71). Qin and his colleagues showed a complete difference between the depression animal model group and the control group related to the expression level of TRPC3/5 and the morphology in neurons, located in the hippocampus (72). Moreover, Buran et al. found that TRPC3/6 inhibitors might play a critical part in the etiopathogenesis of depressive disorders with enhanced levels of miR-9-5p and miR-128-1-5p (73).

### Oxidative Stress (OS)

#### Nuclear Factor Erythroid-2 Related Factor 2 (Nrf2) Pathway

Nrf2 comprises of a basic leucine zipper (bZIP) domain, which plays an important part in regulating the cellular antioxidant defense system. This includes heme oxygenase (HO) and superoxide dismutase (SOD) (74). The functions of Reactive oxygen species (ROS) are to trigger the Keap1/Nrf2/ARE pathways so as to compromise oxidative stress (OS) following ICH, which is known as an adaptive response (75–77). Keap1 is an OS sensor that negatively regulates Nrf2. Upon exposure to ROS, Nrf2 decouples from Keap1 and relocates to the nucleus before activating the antioxidant response element (ARE)-dependent cytoprotective gene that mediates cell survival (78). The neuroprotective effect of Nrf2 suggests that a greater brain damage in Nrf2 knockout mice is correlated with increased ROS and apoptosis (76, 77). Therefore, Nrf2 activation of pharmaceutical preparations is a promising target to alleviate OS-induced brain damage following ICH.

Some researchers have indicated that Nrf2 is a major redox-sensitive transcription factor, which gets involved in the process of cellular self-protection from oxidative damage and increases vulnerability to depression-like actions. As part of a review, depression was characterized by distortion in six interwoven pathways; Maes et al. proposed that inhibitors of the Nrf2 activator target the above six pathways and may produce antidepressant effects (79). Djordjevic et al. revealed the maladaptive characteristics of chronic stress at the Nrf2/Keap1 level, resulted in the production of pro-inflammatory symptoms, suggesting that these changes may take part in the pathogenesis of depression/anxiety disorders (80).

For the past few years, several drugs have been found to have an antidepressant effect by influencing the Nrf2 signaling pathway. Furthermore, their target proteins are expressed in the brain. Mendez-David and colleagues showed that the Nrf2 signaling pathway is necessary for fluoxetine-induced neuroprotection associated with SERT blockade of 5-HT transporters, rather than for enhancing BDNF expression (81). Martín-Hernández et al. confirmed that the Nrf2 pathway is involved in the oxidation/nitrosation damage detected in the prefrontal cortex (PFC); moreover, the antidepressant drug has a therapeutic effect through this route (82). By stimulating PFC, CA3, and TrkB in dentate gyrus in Nrf2-knockout animal experiments, the TrkB agonist, 7,8-dihydroxyflavone, has shown a significant antidepressant functionality (83). Mice pretreated with Nrf2 activator sulforaphane (SFN) revealed reduced depression symptoms, which resulted from frequent social defeat stress. This suggests that the Keap1-Nrf2 interaction has a critical role in the pathophysiology of depression (83). Other Nrf2-activating drugs like TBE-31 and MCE-1 have also been proven as effective for treatment of depression associated to inflammation (84). Agmatine, an endogenous neuromodulator, also promises to serve as adjuvant/monotherapy for depression. This reinforces the importance of antidepressant Nrf2 activators (85). Recently, another drug, cilostazol, manifests promising prophylactic antidepressant-like effect by activating the Nrf2 pathway as well as by recovering mitochondrial malfunction, which interrupts OS (86).

#### PI3K/Akt Pathway

Plenty of brain stroke studies have revealed that ROS/RNS not only directly oxidize cellular macromolecules, such as proteins, lipids, and nucleic acids, which are associated with oxidative damage, but are also involved in the cell apoptosis signaling pathways. The PI3K/Akt, MAPK/P38, and NF-κB pathways are three major OS-mediated pathway activators. Apoptosis mediated by cytochrome c is another important pathway that is mitochondria-dependent (87). In addition, there is growing evidence that the phosphatidylinositol 3-kinase (PI3K)/AKT pathway is associated with the pathophysiology of depression and the antidepressant-like effect of different compounds (88–90).

In recent years, numerous findings have been derived from both basic and clinical researches that suggest erythropoietin has the ability to fight the depressive-like symptoms. Through JAK2, erythropoietin and its receptor signaling activates plenty of downstream signaling pathways such as NF-κB, PI3K/Akt, MAPK, and STAT5, they are able to have a significant role in neuro-progression and inflammation in the CNS (91). Recently, Wu et al. concluded that following the activation and release of neuroinflammatory factor induced by stress, the probable mechanism relates to the idea that the AKT/GSK3β/CRMP-2 pathway changes the normal structure and function of the central nervous cell scaffold microtubule system, and subsequently leads to depression (92). Moreover, Tao et al. proposed that liquiritigenin may reverse depression-like behavior in UCMS-induced animal models by modulating PI3K/Akt/mTOR mediated BDNF/TrkB signaling pathway (93). Several studies have shown that fluoxetine, creatine, atorvastatin, valproic acid as well as IGF-1 can all counteract depression-like behaviors via the PI3K/Akt Pathway (94–99).

#### MAPK/P38 Pathway

Earlier studies have shown that p38 MAPK, which can be stimulated by cytokine, can influence the neuroendocrine function, monoamine neurotransmission as well as other behaviorally-associated pathophysiological pathways (100). Felger et al. indicated that during chronic IFN-α treatment, depressive symptoms are highly related to the sensitivity of the p38 MAPK pathway to immune-stimuli (101). The MAO-A enzyme and p38 MAPK cascade are both involved in OS. These data and *in vitro* experiments demonstrate that the function of MAO-A is strongly inhibited by the p38 MAPK cascade. Thus, these published data indicate that the endogenous approach could be adopted to deal with OS and disorders like depression (102).

Recent research on neuroscience indicates that neurodegenerative pathways and OS pathways are both involved in depression. Bruchas et al. (103) found that the serotonin transporter can translocate to the plasma cell membrane, and that neurotransmitter-uptake is enhanced at the serotonergic nerve terminals when stress induces the activation of p38α MAPK. This finding strongly suggests that a cascade of molecular and cellular events is initiated by stress, and consequently the activation of p38α MAPK leads to a change in the hyposerotonergic state, which underlies drug-seeking and depression-like behaviors (103). MAPK and its phosphatase MKP are found to be implicated in depression and drug-addiction. Findings by Jia et al. supported the idea that there is a direct link between the phosphorylation of MAPK and depression induced by prolonged morphine withdrawal (104). In addition, Park et al. demonstrated that p38 MAPK inhibits the hypoxia response pathway (105). Moreover, Martín-Hernández et al. (106) showed that CMS increased the expression of activated MAPK p38 in addition to decreasing antioxidant transcriptional factor Nrf2. These results suggested that the translocated bacteria played a role through p38 MAPK, which aggravated oxidative injury and neuro-inflammation. This is possibly strongly linked to the pathophysiology of depression (106). These studies indicated an indirect relationship with depression, which requires further research.

Regarding drug therapy, the acute MAPK pathway was blocked, which resulted in depression-like symptoms and prevented the positive effects of ketamine. This fact suggests that the antidepressant response of ketamine is probably regulated by the MAPK pathway in some brain regions (107). Yang et al. reported that fluoxetine (FLX) is able to reduce NF-κB and p38 MAPK phosphorylation levels and may improve the anti-inflammatory consequence (108). Moreover, Moretti et al. extended the data relating to the anti-depressive-like effect of ascorbic acid, which distinctly decreased hippocampal phosphorylation of p38MAPK (109).

### Autophagy

Increased autophagy has now been reported in the central nervous system after several different kinds of diseases, such as ICH. Autophagy is an essential intracellular pathway, which includes degradation and recycling of aged proteins and entire organelles (110, 111). The mTOR pathway, NF-κB pathway and PI3K pathway are major pathways involved in regulating autophagy.

#### The mTOR Signaling Cascade

Mammalian target of rapamycin (mTOR) is a serine/threonine protein kinase that belongs to the phosphoinositide kinase-related kinase family (PIKK family) and is a downstream effector of the PI3K/PKB (protein kinase B) signaling pathway. When its signaling pathway is activated, it has an important presence in regulating protein development, synthesis, proliferation and cell survival. Wang et al. conducted an experiment on mice to try to understand the negative effects of mTOR signaling (and its downstream products) on brain damage that results from ICH. It was found that if mTOR is blocked with rapamycin, PICs, scilicet, TNF-α, IL-6, IL-1β, and Caspase-3 all were upregulated indicating that apoptotic cell death could be reduced remarkably (112).

Several studies have found that ICH upregulated the expression level of miRNA-144 but decreased mTOR expression, which lead to increased inflammation and microglial autophagy. Their findings suggested that miRNA-144 was a critical regulator of autophagy by modulating mTOR (113). Later, another study published by Wang et al. indicated that miRNA-144 contributed to activated autophagy of microglia through the mTOR signaling pathway, which might be mediated by hemoglobin (114). More recently, Shi et al. suggested that IL-17A is a mediator who promoted the activation of inflammation and autophagy in microglial cells (115).

Structural and neurochemical changes in the limbic system are related to depression. The limbic structures include the hippocampus, which plays an important part in controlling emotion and mood. How the mTOR signaling pathway is relating to depression is discussed in many studies. Severe damages in mTOR signaling are revealed in postmortem studies, especially the mTOR signaling that exists in the prefrontal cortex of MDD patients (116). Feng et al. proposed that the depressive disorder is related to PLD-mTOR signaling (117). Lately, studies suggest microRNAs (miRNAs or miRs) such as miR-124-3p are implicated in certain signaling pathways, which might be associated to the pathophysiological mechanism of MDD. It was also suggested that DNA damage-inducible transcript 4 (DDIT4) is an inhibitor of the mammalian target of rapamycin (mTOR) signaling pathway and positively correlates with the expression of miR-124-3p (118).

Recent investigations found that mTOR signaling is related to several types of antidepressant drugs. Yu et al. indicated that the antidepressant effects of ketamine, in patients who have depression, could be reversed by the mTOR signaling inhibitor rapamycin (119). Cui et al. confirmed that by improving plasticity and neurogenesis, the mammalian target of rapamycin (mTOR) signaling pathway has an important role involved in mediating the antidepressant effect of ketamine (120). Nonetheless, drugs such as imipramine are not the same as ketamine, which could inhibit the PI3K/Akt/mTOR signaling to exert its antidepressant effect (116). Moreover, Liu et al. indicated that Resveratrol expresses antidepressant effects in CUMS-induced depressive-like animal models, which was partly mediated by its up-regulation of phosphor-Akt and mTOR expressions in the PFC and hippocampus playing a part in its antioxidant effects (121). Zhang et al. presented a new insight into the role of the dopaminergic system located in mesocortical region, which revealed antidepressant actions during the l-SPD mediated antidepressant process via the D1R/PKA/mTOR signaling cascade in the mPFC (122).

#### NF-κB Pathway

Numerous studies have demonstrated that in several disorders, autophagy is associated with inflammation. Moreover, as a critical controller in inflammation, NF-κB is either mediated by autophagy-related proteins or regulates autophagy directly. Shen et al. indicated that autophagy is activated after ICH, which may exacerbate ICH-induced cerebral damage in animal models. Furthermore, the regulation of the NF-κB pathway maybe a key reason that results in neuro-damage via its promotion of apoptosis and inflammation (123).

As we have described in the former part of this manuscript, the NF-κB pathway is an important pathway that links ICH and ICH-induced depression. Drugs such as Crocin, Icariin, Proanthocyanidin, Senegenin, and others all have antidepressant effects via the NF-κB pathway in ICH-induced depression patients.

### Apoptosis

Study findings suggest that both necrosis and apoptosis following ICH causes cell death. Some experiments revealed that apoptotic cell death existed in brain tissues from both animal models and ICH patients (124, 125). DNA fragmentation and apoptotic cell death are a consequence of activated caspases that are a part of a series of overwhelmingly complicated apoptotic mechanisms. It has been reported that cell death in ICH-induced animal models results from apoptosis mediated by caspases (126, 127). Intrinsic and extrinsic pathways are mainly responsible for apoptosis.

#### Apoptotic Pathways

1. Death receptor-mediated apoptosis pathway—cell death signals are likely initiated by different stimuli, such as tumors, trauma or others. Subsequently, upstream signals bind to Fas-associated proteins that have associated death domains (FADD) and receptors. Then, caspase- 8 is activated via the p53, BCL-2, FAS, NF-κB, and others, which would ultimately lead to the activation of the executioner caspase. The effector caspases then activate endonucleases, resulting in DNA fragmentation, which subsequently orchestrates the dismantling of the whole cell structure.
2. Mitochondrial apoptosis pathway—often regulated by B-cell lymphoma-2 (BCL-2) family protein. As a trigger, intrinsic signals could inhibit the pro-apoptotic BCL-2 family protein and deactivate the anti-apoptotic function of BCL-2. Consequently, the mitochondria will release cytochrome c abundantly into the cytosol, which is a significant component of the complex, apoptotic protease activating factor-1 (APAF-1) and pro-caspase-9. Downstream effector molecules activated by the Cyt-c-Apaf- 1- Procaspase- 9 complex result in apoptosis.
3. Caspase-independent pathways—Apoptosis-inducing factor (AIF), as an intermembrane protein of mitochondrial, is regulated by p53 in the absent of APAF-I, and activated by the caspase-independent pathways.

Finally, together, procaspase-8,−9, cytochrome c and other signal proteins constitute the “apoptosome,” which activates the initiator caspases such as caspase 8 and−9. After that, either the extrinsic or intrinsic apoptosis pathway delivers the cell death signal to the final executioner caspase (caspase-3,−6,−7) and subsequently initiates enzymes that degrade DNA, RNA and ribose. After the process of activating procaspase to caspase, programmed cell death is initiated (128).

Depression is a condition related to abnormal brain energy metabolism that is also marked with increased apoptosis in specific cerebral areas. Bay 60-7550 (Phosphodiesterase 2 inhibitors) has been shown to be a mediator in the apoptotic process, possibly via the SOD-cGMP/PKG-anti-apoptosis signaling pathway in neuronal cells, and by inhibiting PDE2; it may be a significant novel antidepressant therapy (129). Moreover, water extracted from Panax ginseng (WEG) has been used as a treatment of several CNS disorders. Ding et al. suggested previously that WEG performed antidepressant-like effects in animal models of depression that was treated for both chronic and acute stress. Its neuroprotective effect relies on corticosterone-induced apoptosis via the downregulation of cytochrome C, ICAD, caspase-3, caspase-9 and so on (130). Moreover, both risperidone, at medium dose, and paroxetine were reported to improve modified stress re-stress (SRS)-induced depressive- like behaviors with associated down-regulated levels of cytochrome-C and caspase-9 in several regions of the brain (131). In addition, a novel antidepressant drug, Agomelatine (AG), might play an important part in the pathophysiology of depression with the amelioration of the apoptotic cells and the increase of neurogenesis in the hippocampus (132). Moreover, Apocynum venetum leaf extract (AVLE) was also reported to exert antidepressant-like activities in CUMS-induced rat models, which possibly suppressed neuronal apoptosis by regulating the Bcl-2/Bax signaling pathways, and improved the BDNF expressions in the hippocampus (133). Overload of Ca^2+^ entry as well as excessive OS in neurons are the **two** main causes of depression. Recently, Demirdaş et al. reported that with the treatment of Duloxetine (DULOX), TRPM2 and TRPV1 channels (associated with Ca^2+^ entry-induced neuronal death), were regulated to reduce apoptosis in depression-like rats models (134).

#### Other Pathways

Pannexins serves a significant role in the regulation of cellular signal transduction of glial cells and extracellular neuronal regenerative currents. Nevertheless, there have been no reported findings regarding the effects of pannexins in various cerebrovascular diseases. Zhou et al. first suggested that the upregulation of Pannexin-1 (Panx1) expression may be correlated with degeneration and apoptotic cell death of neurons in the rat cerebrum after ICH. Furthermore, he speculated that this may lead to subsequent cognitive dysfunction (135). Recently, Ni et al. used a broad-spectrum Panx1 inhibitor called Mefloquine (MFQ), demonstrating that the Panx1 channel played an important role in chronic stress and MFQ-induced depression and anxiety behaviors (136).

Recently, NIX was elucidated as a novel p75 neurotrophin receptor (p75^NTR^) binding protein as well as a member of the pro-apoptotic BH3-only group of proteins. When exposed to glutamate, the connection between NIX and p75^NTR^, there was marked increase in the apoptosis of neurons and activation of the JNK-p53-Bax pathway (137). Fujii et al. previously offered verification for the connection between the Ser205Leu polymorphism of the p75(NTR) gene as well as MDD, which indicated that the Leu205 allele provides a protective influence to fight the development of MDD (138).

In addition, Zhou et al. suggested that the Wnt/β-catenin signaling pathway is related to the level of proliferating cell nuclear antigen (PCNA) that is present in the cerebrum of the ICH rat, in addition to the rate of cell apoptosis, it could even regulate the balance of cell proliferation and apoptosis (139). Furthermore, Ni et al. (136) found that lncRNA TCONS_00019174 exerts an antidepressant effect in rats by activating the Wnt/β-catenin pathway (139).

These new signaling pathways are proposed as potential clinical therapeutic targets for depressive disorders. This may require further research in order to explore further the relationships between ICH and depression.

## Conclusions

The pathophysiology of PSD is extremely complex; A multitude of ischemia-induced neurobiological mal-function as well as psychosocial distress are involved. The symptom for alterations of monoaminergic neurotransmitter systems has been well presented due to the injury of frontal-basal ganglia brainstem pathway. It has also been proved that there is a strong relationship between neuroinflammation and acute ischemic stroke: stress-induced activation of the hypothalamic-pituitary-adrenal (HPA) axis and the deficit of adaptive response (140).

In this review, we addressed the mechanisms and therapeutic targets of post-ICH depression. We divided the mechanisms into inflammation, oxidative stress, autophagy, and apoptosis, and clarified them through several signaling pathways. Inflammation is mainly related to TLRs, NF-κB mediated signal pathway, the PPAR-γ-dependent pathway and other signaling pathways. OS is related to Nrf2, the PI3K/Akt pathway and the MAPK/P38 pathway. Autophagy is associated with the mTOR signaling cascade and NF-kB mediated signal pathway. Meanwhile, apoptosis is related to the death receptor-mediated apoptosis pathway, mitochondrial apoptosis pathway, caspase-independent pathways as well as other pathways. Based on the evidence listed above, we found that neuroinflammation, OS, autophagy and apoptosis interacted with each other. OS-related brain injury is part of the pathogenic mechanism of neutrophil infiltration that follows ICH (16). Inducible NOS (iNOS) is synthesized through the induction of proinflammatory cytokines, and the molecular mechanisms for NOS activation after ICH are primarily NF-kB dependent (141, 142). If NF-κB and antioxidative defense components can be inhibited, then OS and inflammation can be reduced via PPARγ; in the meantime, the cerebral damage caused by ICH would be improved. Proinflammatory cytokines, namely TNF-α and IL-1, could induce iNOS expression in microglial cells via the KC/p38MAFP/NF-kB pathway (143). Free radicals can also induce apoptosis, and antioxidant therapy could alleviate neuronal apoptosis after ICH (144, 145). The NF-kB pathway has also been detected to mediate Hb-induced apoptosis and autophagy (146). mTOR, as a downstream effector of the PI3K/PKB signaling pathway, also plays a significant part in CNS apoptosis and autophagy. Interactions of TLRs with pathogen-associated molecular patterns (PAMP) and damage-associated molecular patterns (DAMP) initiate signaling through myeloid differentiation primary response-88 (MyD88) and produce cytokines through the activation of the transcription factor nuclear factor kappa beta (NF-kB) (147). Furthermore, PPARγ could stimulate hematoma regression mediated by phagocytosis, and facilitate the cleanup of the hematomas, which may reduce the generation of inflammation and toxicity. Overall, from the assessed antidepressant drugs for ICH-induced depression, we found that several drugs exerted their antidepressant-like effects via different signaling pathways and may have different pathophysiological origins (e.g., ketamine could treat depression through mTOR signaling cascade, the MAPK/P38 pathway or TLR-related signal pathways). This could provide us with evidence that some underlying correlations may exist between different signaling pathways. However, this still requires more research.

In summary, our review presents the signaling pathways relevant to post-ICH depression. Additionally, it may provide several potential therapeutic targets for the treatment of patients who show depressive behavior after ICH (Table 1).

**Table 1:** The relationship among pathophysiology of ICH-induced depression, underlying signal pathways and its potential antidepressant drugs.

**Pathophysiology of ICH-induced depression**	**Signal pathways**	**Antidepressant drugs**
Inflammation	Toll-like receptors (18, 19)	Tianeptine (29), Ketamine (30), Fluoxetine (27), TDZD-8 (27)
	NF-kB mediated signal pathway (36, 37)	Crocin (45), Proanthocyanidin (46), Senegenin (47), Icariin (48), Geraniol (49), Chrysophanol (50)
	PPAR-γ-dependent pathway (54, 55)	Pioglitazone (60), Astragaloside IV (61), Rosiglitazone (64), Troglitazone (67), Atorvastatin (68), Folic acid (69)
	other signaling pathways (71)	TRPC3/6 inhibitor (73)
Oxidative stress	Nrf2 pathway (74–77)	Fluoxetine (81), 7,8-dihydroxyflavone (83), Sulforaphane (83), TBE-31 (84), MCE-1 (84), Agmatine (85), Cilostazol (86)
	the PI3K/Akt pathway (88–90)	Erythropoietin (91), Liquiritigenin (93), Fluoxetine (94), Creatine (95, 96), Atorvastatin (97), valproic acid (98), IGF-1 (99)
	the MAPK/P38 pathway (100)	Ketamine (107), Fluoxetine (108), Ascorbic acid (109)
Autophagy	mTOR signaling cascade (112)	Ketamine (119, 120), Imipramine (116), Resveratrol (121)
	NF-kB mediated signal pathway (123)	Crocin (45), Proanthocyanidin (46), Senegenin (47), Icariin (48), Geraniol (49), Chrysophanol (50)
	the PI3K/Akt pathway (116)	Erythropoietin (91), Liquiritigenin (93), Fluoxetine (94), Creatine (95, 96), Atorvastatin (97), valproic acid (98), IGF-1 (99)
Apoptosis	Death receptor-mediated apoptosis pathway Mitochondrial apoptosis pathway Caspase-independent pathways	Bay 60-7550 (129), Water extracted from Panax ginseng (130), Risperidone (131), Paroxetine (131), Agomelatine (132), Apocynum venetum leaf extract (133), Duloxetine (134)
	other signaling pathways (135, 137, 139)	Mefloquine (136), lncRNA TCONS_00019174 (139)

Depression has a complex relevance with enhanced mortality and morbidity in ICH patients. In spite of its great clinical evidence, the underlying etiological mechanisms are still worthy to be explored. To better understand its pathophysiology and to pursue a more promising outcome of post-ICH depression, therapeutic interventions have become progressively more important for future studies.

## Author Contributions

All authors participated in designing the concept of this manuscript. YW, LW, CY, and KH reviewed the literature and drafted the article. YZ, SZ, and AS finalized the paper and provided suggestions to improve it.

### Conflict of Interest Statement

The authors declare that the research was conducted in the absence of any commercial or financial relationships that could be construed as a potential conflict of interest.

## References

[1] Benjamin EJ Virani SS Callaway CW Chamberlain AM Chang AR Cheng S. Heart disease and stroke statistics-2018 update: a report from the american heart association. Circulation (2018) 137:e67–492. 10.1161/CIR.0000000000000558 29386200

[2] Gilsanz P Walter S Tchetgen Tchetgen EJ Patton KK Moon JR Capistrant BD. Changes in depressive symptoms and incidence of first stroke among middle-aged and older US adults. J Am Heart Assoc. (2015) 4:e001923. 10.1161/JAHA.115.001923 25971438 PMC4599421

[3] Kim YR Kim HN Pak ME Ahn SM Hong KH Shin HK. Studies on the animal model of post-stroke depression and application of antipsychotic aripiprazole. Behav Brain Res. (2015) 287:294–303. 10.1016/j.bbr.2015.03.062 25845738

[4] Lenzi GL Altieri M Maestrini I. Post-stroke depression. Rev Neurol. (2008) 164:837–40. 10.1016/j.neurol.2008.07.010 18771785

[5] Koivunen RJ Harno H Tatlisumak T Putaala J. Depression, anxiety, and cognitive functioning after intracerebral hemorrhage. Acta Neurol Scand. (2015) 132:179–84. 10.1111/ane.12367 25639837

[6] Stern-Nezer S Eyngorn I Mlynash M Snider RW Venkatsubramanian C Wijman CAC. Depression one year after hemorrhagic stroke is associated with late worsening of outcomes. NeuroRehabilitation (2017) 41:179–87. 10.3233/NRE-171470 28505996

[7] Mayer SA Rincon F. Treatment of intracerebral haemorrhage. Lancet Neurol. (2005) 4:662–72. 10.1016/S1474-4422(05)70195-2 16168935

[8] Aronowski J Zhao X. Molecular pathophysiology of cerebral hemorrhage: secondary brain injury. Stroke (2011) 42:1781–6. 10.1161/STROKEAHA.110.596718 21527759 PMC3123894

[9] Babu R Bagley JH Di C Friedman AH Adamson C. Thrombin and hemin as central factors in the mechanisms of intracerebral hemorrhage-induced secondary brain injury and as potential targets for intervention. Neurosurg Focus (2012) 32:E8. 10.3171/2012.1.FOCUS11366 22463118

[10] Elliott J Smith M. The acute management of intracerebral hemorrhage: a clinical review. Anesth Analg. (2010) 110:1419–27. 10.1213/ANE.0b013e3181d568c8 20332192

[11] Felberg RA Grotta JC Shirzadi AL Strong R Narayana P Hill-Felberg SJ. Cell death in experimental intracerebral hemorrhage: the “black hole” model of hemorrhagic damage. Ann Neurol. (2002) 51:517–24. 10.1002/ana.10160 11921058

[12] Huang FP Xi G Keep RF Hua Y Nemoianu A Hoff JT. Brain edema after experimental intracerebral hemorrhage: role of hemoglobin degradation products. J Neurosurg. (2002) 96:287–93. 10.3171/jns.2002.96.2.0287 11838803

[13] Lee KR Kawai N Kim S Sagher O Hoff JT. Mechanisms of edema formation after intracerebral hemorrhage: effects of thrombin on cerebral blood flow, blood-brain barrier permeability, and cell survival in a rat model. J Neurosurg. (1997) 86:272–8. 10.3171/jns.1997.86.2.0272 9010429

[14] Keep RF Hua Y Xi G. Intracerebral haemorrhage: mechanisms of injury and therapeutic targets. Lancet Neurol. (2012) 11:720–31. 10.1016/S1474-4422(12)70104-7 22698888 PMC3884550

[15] Wang J Dore S. Inflammation after intracerebral hemorrhage. J Cereb Blood Flow Metab. (2007) 27:894–908. 10.1038/sj.jcbfm.9600403 17033693

[16] Wang J. Preclinical and clinical research on inflammation after intracerebral hemorrhage. Prog Neurobiol. (2010) 92:463–77. 10.1016/j.pneurobio.2010.08.001 20713126 PMC2991407

[17] Xi G Keep RF Hoff JT. Mechanisms of brain injury after intracerebral haemorrhage. Lancet Neurol. (2006) 5:53–63. 10.1016/S1474-4422(05)70283-0 16361023

[18] Akira S Takeda K Toll-like receptor signalling. Nat Rev Immunol. (2004) 4:499–511. 10.1038/nri1391 15229469

[19] Ransohoff RM Brown MA. Innate immunity in the central nervous system. J Clin Invest. (2012) 122:1164–71. 10.1172/JCI58644 22466658 PMC3314450

[20] Sansing LH Harris TH Welsh FA Kasner SE Hunter CA Kariko K. Toll-like receptor 4 contributes to poor outcome after intracerebral hemorrhage. Ann Neurol. (2011) 70:646–56. 10.1002/ana.22528 22028224 PMC3671585

[21] Rodriguez-Yanez M Brea D Arias S Blanco M Pumar JM Castillo J. Increased expression of Toll-like receptors 2 and 4 is associated with poor outcome in intracerebral hemorrhage. J Neuroimmunol. (2012) 247:75–80. 10.1016/j.jneuroim.2012.03.019 22498099

[22] Lin S Yin Q Zhong Q Lv FL Zhou Y Li JQ. Heme activates TLR4-mediated inflammatory injury via MyD88/TRIF signaling pathway in intracerebral hemorrhage. J Neuroinflamm. (2012) 9:46. 10.1186/1742-2094-9-46 22394415 PMC3344687

[23] Fang H Wang PF Zhou Y Wang YC Yang QW. Toll-like receptor 4 signaling in intracerebral hemorrhage-induced inflammation and injury. J Neuroinflamm. (2013) 10:27. 10.1186/1742-2094-10-27 23414417 PMC3598479

[24] Keri S Szabo C Kelemen O. Expression of toll-like receptors in peripheral blood mononuclear cells and response to cognitive-behavioral therapy in major depressive disorder. Brain Behav Immun. (2014) 40:235–43. 10.1016/j.bbi.2014.03.020 24726793

[25] Strekalova T Evans M Costa-Nunes J Bachurin S Yeritsyan N Couch Y. Tlr4 upregulation in the brain accompanies depression- and anxiety-like behaviors induced by a high-cholesterol diet. Brain Behav Immun. (2015) 48:42–7. 10.1016/j.bbi.2015.02.015 25712260

[26] Habib M Shaker S El-Gayar N Aboul-Fotouh S The effects of antidepressants “fluoxetine and imipramine” on vascular abnormalities and Toll like receptor-4 expression in diabetic and non-diabetic rats exposed to chronic stress. PLoS ONE (2015) 10:e0120559 10.1371/journal.pone.0120559 25826421 PMC4380417

[27] Cheng Y Pardo M Armini RS Martinez A Mouhsine H Zagury JF. Stress-induced neuroinflammation is mediated by GSK3-dependent TLR4 signaling that promotes susceptibility to depression-like behavior. Brain Behav Immun. (2016) 53:207–222. 10.1016/j.bbi.2015.12.012 26772151 PMC4783243

[28] Garcia Bueno B Caso JR Madrigal JL Leza JC Innate immune receptor Toll-like receptor 4 signalling in neuropsychiatric diseases. Neurosci Biobehav Rev. (2016) 64:134–47. 10.1016/j.neubiorev.2016.02.013 26905767

[29] Slusarczyk J Trojan E Glombik K Piotrowska A Budziszewska B Kubera M. Targeting the NLRP3 inflammasome-related pathways via tianeptine treatment-suppressed microglia polarization to the M1 phenotype in lipopolysaccharide-stimulated cultures. Int J Mol Sci. (2018) 19:E1965. 10.3390/ijms19071965 29976873 PMC6073715

[30] Tan S Wang Y Chen K Long Z Zou J. Ketamine alleviates depressive-like behaviors via down-regulating inflammatory cytokines induced by chronic restraint stress in mice. Biol Pharm Bull. (2017) 40:1260–7. 10.1248/bpb.b17-00131 28769008

[31] Beurel E. Regulation by glycogen synthase kinase-3 of inflammation and T cells in CNS diseases. Front Mol Neurosci. (2011) 4:18. 10.3389/fnmol.2011.00018 21941466 PMC3171068

[32] Beurel E Grieco SF Jope RS. Glycogen synthase kinase-3 (GSK3): regulation, actions, and diseases. Pharmacol Ther. (2015) 148:114–31. 10.1016/j.pharmthera.2014.11.016 25435019 PMC4340754

[33] Martin M Rehani K Jope RS Michalek SM. Toll-like receptor-mediated cytokine production is differentially regulated by glycogen synthase kinase 3. Nat Immunol. (2005) 6:777–84. 10.1038/ni1221 16007092 PMC1933525

[34] Yuskaitis CJ Jope RS. Glycogen synthase kinase-3 regulates microglial migration, inflammation, and inflammation-induced neurotoxicity. Cell Signal (2009) 21:264–73. 10.1016/j.cellsig.2008.10.014 19007880 PMC2630396

[35] Beurel E Jope RS. Lipopolysaccharide-induced interleukin-6 production is controlled by glycogen synthase kinase-3 and STAT3 in the brain. J Neuroinflamm. (2009) 6:9. 10.1186/1742-2094-6-9 19284588 PMC2660311

[36] Aronowski J Hall CE. New horizons for primary intracerebral hemorrhage treatment: experience from preclinical studies. Neurol Res. (2005) 27:268–79. 10.1179/016164105X25225 15845210

[37] Wagner KR. Modeling intracerebral hemorrhage: glutamate, nuclear factor-kappa B signaling and cytokines. Stroke (2007) 38 (Suppl. 2):753–8. 10.1161/01.STR.0000255033.02904.db 17261732

[38] Su X Wang H Zhu L Zhao J Pan H Ji X. Ethyl pyruvate ameliorates intracerebral hemorrhage-induced brain injury through anti-cell death and anti-inflammatory mechanisms. Neuroscience (2013) 245:99–108. 10.1016/j.neuroscience.2013.04.032 23624063

[39] Koo JW Russo SJ Ferguson D Nestler EJ Duman RS. Nuclear factor-kappaB is a critical mediator of stress-impaired neurogenesis and depressive behavior. Proc Natl Acad Sci USA. (2010) 107:2669–74. 10.1073/pnas.0910658107 20133768 PMC2823860

[40] Lukic I Mitic M Djordjevic J Tatalovic N Bozovic N Soldatovic I. Lymphocyte levels of redox-sensitive transcription factors and antioxidative enzymes as indicators of pro-oxidative state in depressive patients. Neuropsychobiology (2014) 70:1–9. 10.1159/000362841 25170744

[41] Bortolotto V Cuccurazzu B Canonico PL Grilli M. NF-kappaB mediated regulation of adult hippocampal neurogenesis: relevance to mood disorders and antidepressant activity. Biomed Res Int. (2014) 2014:612798. 10.1155/2014/612798 24678511 PMC3942292

[42] Nadeem A Ahmad SF Al-Harbi NO Fardan AS El-Sherbeeny AM Ibrahim KE. IL-17A causes depression-like symptoms via NFkappaB and p38MAPK signaling pathways in mice: implications for psoriasis associated depression. Cytokine (2017) 97:14–24. 10.1016/j.cyto.2017.05.018 28570931

[43] Kim TK Kim JE Choi J Park JY Lee JE Lee EH Local interleukin-18 system in the basolateral amygdala regulates susceptibility to chronic stress. Mol Neurobiol. (2017) 54:5347–58. 10.1007/s12035-016-0052-7 27590137

[44] Su WJ Zhang Y Chen Y Gong H Lian YJ Peng W. NLRP3 gene knockout blocks NF-kappaB and MAPK signaling pathway in CUMS-induced depression mouse model. Behav Brain Res. (2017) 322 (Pt A):1–8. 10.1016/j.bbr.2017.01.018 28093255

[45] Zhang L Previn R Lu L Liao RF Jin Y Wang RK. Crocin, a natural product attenuates lipopolysaccharide-induced anxiety and depressive-like behaviors through suppressing NF-kB and NLRP3 signaling pathway. Brain Res Bull. (2018) 142:352–9. 10.1016/j.brainresbull.2018.08.021 30179677

[46] Jiang X Liu J Lin Q Mao K Tian F Jing C. Proanthocyanidin prevents lipopolysaccharide-induced depressive-like behavior in mice via neuroinflammatory pathway. Brain Res Bull. (2017) 135:40–46. 10.1016/j.brainresbull.2017.09.010 28941603

[47] Li H Lin S Qin T Li H Ma Z Ma S. Senegenin exerts anti-depression effect in mice induced by chronic un-predictable mild stress via inhibition of NF-kappaB regulating NLRP3 signal pathway. Int Immunopharmacol. (2017) 53:24–32. 10.1016/j.intimp.2017.10.001 29031144

[48] Liu B Xu C Wu X Liu F Du Y Sun J. Icariin exerts an antidepressant effect in an unpredictable chronic mild stress model of depression in rats and is associated with the regulation of hippocampal neuroinflammation. Neuroscience (2015) 294:193–205. 10.1016/j.neuroscience.2015.02.053 25791226

[49] Deng XY Xue JS Li HY Ma ZQ Fu Q Qu R. Geraniol produces antidepressant-like effects in a chronic unpredictable mild stress mice model. Physiol Behav. (2015) 152 (Pt A):264–71. 10.1016/j.physbeh.2015.10.008 26454213

[50] Zhang K Liu J You X Kong P Song Y Cao L. P2X7 as a new target for chrysophanol to treat lipopolysaccharide-induced depression in mice. Neurosci Lett, (2016) 613:60–5. 10.1016/j.neulet.2015.12.043 26724370

[51] Baranova IN Kurlander R Bocharov AV Vishnyakova TG Chen Z Remaley AT. Role of human CD36 in bacterial recognition, phagocytosis, and pathogen-induced JNK-mediated signaling. J Immunol. (2008) 181:7147–56. 10.4049/jimmunol.181.10.7147 18981136 PMC3842223

[52] Fadok VA Warner ML Bratton DL Henson PM CD36 is required for phagocytosis of apoptotic cells by human macrophages that use either a phosphatidylserine receptor or the vitronectin receptor (alpha v beta 3). J Immunol. (1998) 161:6250–7. 9834113

[53] Zeng Y Tao N Chung KN Heuser JE Lublin DM Endocytosis of oxidized low density lipoprotein through scavenger receptor CD36 utilizes a lipid raft pathway that does not require caveolin-1. J Biol Chem. (2003) 278:45931–6. 10.1074/jbc.M307722200 12947091

[54] Sundararajan S Gamboa JL Victor NA Wanderi EW Lust WD Landreth GE. Peroxisome proliferator-activated receptor-gamma ligands reduce inflammation and infarction size in transient focal ischemia. Neuroscience (2005) 130:685–96. 10.1016/j.neuroscience.2004.10.021 15590152

[55] Tontonoz P Nagy L Alvarez JG Thomazy VA Evans RM. PPARgamma promotes monocyte/macrophage differentiation and uptake of oxidized LDL. Cell (1998) 93:241–52. 10.1016/S0092-8674(00)81575-5 9568716

[56] Flores JJ Klebe D Rolland WB Lekic T Krafft PR Zhang JH. PPARgamma-induced upregulation of CD36 enhances hematoma resolution and attenuates long-term neurological deficits after germinal matrix hemorrhage in neonatal rats. Neurobiol Dis. (2016) 87:124–33. 10.1016/j.nbd.2015.12.015 26739391 PMC4724557

[57] Gold PW Licinio J Pavlatou MG. Pathological parainflammation and endoplasmic reticulum stress in depression: potential translational targets through the CNS insulin, klotho and PPAR-gamma systems. Mol Psychiatry (2013) 18:154–65. 10.1038/mp.2012.167 23183489 PMC10064987

[58] Agudelo LZ Femenia T Orhan F Porsmyr-Palmertz M Goiny M Martinez-Redondo V. Skeletal muscle PGC-1alpha1 modulates kynurenine metabolism and mediates resilience to stress-induced depression. Cell (2014) 159:33–45. 10.1016/j.cell.2014.07.051 25259918

[59] Colle R de Larminat D Rotenberg S Hozer F Hardy P Verstuyft C. PPAR-gamma agonists for the treatment of major depression: a review. Pharmacopsychiatry (2017) 50:49–55. 10.1055/s-0042-120120 27978584

[60] Liao L Zhang XD Li J Zhang ZW Yang CC Rao CL Zhou CJ. Pioglitazone attenuates lipopolysaccharide-induced depression-like behaviors, modulates NF-kappaB/IL-6/STAT3, CREB/BDNF pathways and central serotonergic neurotransmission in mice. Int Immunopharmacol. (2017) 49:178–86. 10.1016/j.intimp.2017.05.036 28595081

[61] Song MT Ruan J Zhang RY Deng J Ma ZQ Ma SP. Astragaloside IV ameliorates neuroinflammation-induced depressive-like behaviors in mice via the PPARgamma/NF-kappaB/NLRP3 inflammasome axis. Acta Pharmacol Sin. (2018) 39:1559–70. 10.1038/aps.2017.208 29795356 PMC6289360

[62] Zeinoddini A Sorayani M Hassanzadeh E Arbabi M Farokhnia M Salimi S. Pioglitazone adjunctive therapy for depressive episode of bipolar disorder: a randomized, double-blind, placebo-controlled trial. Depress Anxiety (2015) 32:167–73. 10.1002/da.22340 25620378

[63] Bonato JM Bassani TB Milani H Vital M de Oliveira RMW. Pioglitazone reduces mortality, prevents depressive-like behavior, and impacts hippocampal neurogenesis in the 6-OHDA model of Parkinson's disease in rats. Exp Neurol. (2018) 300:188–200. 10.1016/j.expneurol.2017.11.009 29162435

[64] Eissa Ahmed AA Al-Rasheed NM Al-Rasheed NM. Antidepressant-like effects of rosiglitazone, a PPARgamma agonist, in the rat forced swim and mouse tail suspension tests. Behav Pharmacol. (2009) 20:635–42. 10.1097/FBP.0b013e328331b9bf 19745723

[65] Zong J Liao X Ren B Wang Z. The antidepressant effects of rosiglitazone on rats with depression induced by neuropathic pain. Life Sci. (2018) 203:315–22. 10.1016/j.lfs.2018.04.057 29730170

[66] Zhao Z Zhang L Guo XD Cao LL Xue TF Zhao XJ. Rosiglitazone exerts an anti-depressive effect in unpredictable chronic mild-stress-induced depressive mice by maintaining essential neuron autophagy and inhibiting excessive astrocytic apoptosis. Front Mol Neurosci. (2017) 10:293. 10.3389/fnmol.2017.00293 28959186 PMC5603714

[67] d'Abramo C Ricciarelli R Pronzato MA Davies P. Troglitazone, a peroxisome proliferator-activated receptor-gamma agonist, decreases tau phosphorylation in CHOtau4R cells. J Neurochem. (2006) 98:1068–77. 10.1111/j.1471-4159.2006.03931.x 16787414

[68] Shahsavarian A Javadi S Jahanabadi S Khoshnoodi M Shamsaee J Shafaroodi H. Antidepressant-like effect of atorvastatin in the forced swimming test in mice: the role of PPAR-gamma receptor and nitric oxide pathway. Eur J Pharmacol. (2014) 745:52–8. 10.1016/j.ejphar.2014.10.004 25446923

[69] Budni J Lobato KR Binfare RW Freitas AE Costa AP Martin-de-Saavedra MD. Involvement of PI3K, GSK-3beta and PPARgamma in the antidepressant-like effect of folic acid in the forced swimming test in mice. J Psychopharmacol. (2012) 26:714–23. 10.1177/0269881111424456 22037925

[70] Naziroglu M Demirdas A. Psychiatric disorders and TRP channels: focus on psychotropic drugs. Curr Neuropharmacol. (2015) 13:248–57. 10.2174/1570159X13666150304001606 26411768 PMC4598437

[71] Mizoguchi Y Monji A. TRPC channels and brain inflammation. Adv Exp Med Biol. (2017) 976:111–121. 10.1007/978-94-024-1088-4_10 28508317

[72] Qin X Liu Y Zhu M Yang Z. The possible relationship between expressions of TRPC3/5 channels and cognitive changes in rat model of chronic unpredictable stress. Behav Brain Res. (2015) 290:180–6. 10.1016/j.bbr.2015.04.054 25958233

[73] Buran I Etem EO Tektemur A Elyas H. Treatment with TREK1 and TRPC3/6 ion channel inhibitors upregulates microRNA expression in a mouse model of chronic mild stress. Neurosci Lett. (2017) 656:51–7. 10.1016/j.neulet.2017.07.017 28716528

[74] Zhao X Aronowski J. Nrf2 to pre-condition the brain against injury caused by products of hemolysis after ICH. Transl Stroke Res. (2013) 4:71–5. 10.1007/s12975-012-0245-y 23378859 PMC3558942

[75] Shang H Yang D Zhang W Li T Ren X Wang X. Time course of Keap1-Nrf2 pathway expression after experimental intracerebral haemorrhage: correlation with brain oedema and neurological deficit. Free Radic Res. (2013) 47:368–75. 10.3109/10715762.2013.778403 23438812

[76] Wang J Fields J Zhao C Langer J Thimmulappa RK Kensler TW. Role of Nrf2 in protection against intracerebral hemorrhage injury in mice. Free Radic Biol Med. (2007) 43:408–14. 10.1016/j.freeradbiomed.2007.04.020 17602956 PMC2039918

[77] Zhao X Sun G Zhang J Strong R Dash PK Kan YW. Transcription factor Nrf2 protects the brain from damage produced by intracerebral hemorrhage. Stroke (2007) 38:3280–6. 10.1161/STROKEAHA.107.486506 17962605

[78] Qaisiya M Coda Zabetta CD Bellarosa C Tiribelli C. Bilirubin mediated oxidative stress involves antioxidant response activation via Nrf2 pathway. Cell Signal (2014) 26:512–20. 10.1016/j.cellsig.2013.11.029 24308969

[79] Maes M Fisar Z Medina M Scapagnini G Nowak G Berk M. New drug targets in depression: inflammatory, cell-mediated immune, oxidative and nitrosative stress, mitochondrial, antioxidant, and neuroprogressive pathways. And new drug candidates–Nrf2 activators and GSK-3 inhibitors. Inflammopharmacology (2012) 20:127–50. 10.1007/s10787-011-0111-7 22271002

[80] Djordjevic J Djordjevic A Adzic M Mitic M Lukic I Radojcic MB. Alterations in the Nrf2-Keap1 signaling pathway and its downstream target genes in rat brain under stress. Brain Res. (2015) 1602:20–31. 10.1016/j.brainres.2015.01.010 25598205

[81] Mendez-David I Tritschler L Ali ZE Damiens MH Pallardy M David DJ. Nrf2-signaling and BDNF: a new target for the antidepressant-like activity of chronic fluoxetine treatment in a mouse model of anxiety/depression. Neurosci Lett. (2015) 597:121–6. 10.1016/j.neulet.2015.04.036 25916883

[82] Martin-Hernandez D Bris AG MacDowell KS Garcia-Bueno B Madrigal JL Leza JC. Modulation of the antioxidant nuclear factor (erythroid 2-derived)-like 2 pathway by antidepressants in rats. Neuropharmacology (2016) 103:79–91. 10.1016/j.neuropharm.2015.11.029 26686388

[83] Yao W Zhang JC Ishima T Dong C Yang C Ren Q. Role of Keap1-Nrf2 signaling in depression and dietary intake of glucoraphanin confers stress resilience in mice. Sci Rep. (2016) 6:30659. 10.1038/srep30659 27470577 PMC4965765

[84] Yao W Zhang JC Ishima T Ren Q Yang C Dong C. Antidepressant effects of TBE-31 and MCE-1, the novel Nrf2 activators, in an inflammation model of depression. Eur J Pharmacol. (2016) 793:21–7. 10.1016/j.ejphar.2016.10.037 27815170

[85] Freitas AE Egea J Buendia I Gomez-Rangel V Parada E Navarro E. Agmatine, by improving neuroplasticity markers and inducing Nrf2, prevents corticosterone-induced depressive-like behavior in mice. Mol Neurobiol. (2016) 53:3030–45. 10.1007/s12035-015-9182-6 25966970

[86] Abuelezz SA Hendawy N. Insights into the potential antidepressant mechanisms of cilostazol in chronically restraint rats: impact on the Nrf2 pathway. Behav Pharmacol. (2018) 29:28–40. 10.1097/FBP.0000000000000335 28763303

[87] Chan PH. Mitochondria and neuronal death/survival signaling pathways in cerebral ischemia. Neurochem Res. (2004) 29:1943–9. 10.1007/s11064-004-6869-x 15662830

[88] Kitagishi Y Kobayashi M Kikuta K Matsuda S. Roles of PI3K/AKT/GSK3/mTOR pathway in cell signaling of mental illnesses. Depress Res Treat. (2012) 2012:752563. 10.1155/2012/752563 23320155 PMC3535741

[89] Shi HS Zhu WL Liu JF Luo YX Si JJ Wang SJ. PI3K/Akt signaling pathway in the basolateral amygdala mediates the rapid antidepressant-like effects of trefoil factor 3. Neuropsychopharmacology (2012) 37:2671–83. 10.1038/npp.2012.131 22828749 PMC3473333

[90] Numakawa T Adachi N Richards M Chiba S Kunugi H. Brain-derived neurotrophic factor and glucocorticoids: reciprocal influence on the central nervous system. Neuroscience (2013) 239:157–72. 10.1016/j.neuroscience.2012.09.073 23069755

[91] Ma C Cheng F Wang X Zhai C Yue W Lian Y. Erythropoietin pathway: a potential target for the treatment of depression. Int J Mol Sci. (2016) 17:E677. 10.3390/ijms17050677 27164096 PMC4881503

[92] Wu Z Wang G Wei Y Xiao L Wang H. PI3K/AKT/GSK3beta/CRMP-2-mediated neuroplasticity in depression induced by stress. Neuroreport (2018) 29:1256–63. 10.1097/WNR.0000000000001096 30113922

[93] Tao W Dong Y Su Q Wang H Chen Y Xue W. Liquiritigenin reverses depression-like behavior in unpredictable chronic mild stress-induced mice by regulating PI3K/Akt/mTOR mediated BDNF/TrkB pathway. Behav Brain Res. (2016) 308:177–86. 10.1016/j.bbr.2016.04.039 27113683

[94] Zeng B Li Y Niu B Wang X Cheng Y Zhou Z. Involvement of PI3K/Akt/FoxO3a and PKA/CREB signaling pathways in the protective effect of fluoxetine against corticosterone-induced cytotoxicity in PC12 cells. J Mol Neurosci. (2016) 59:567–78. 10.1007/s12031-016-0779-7 27412469

[95] Pazini FL Cunha MP Rosa JM Colla AR Lieberknecht V Oliveira A. Creatine, similar to ketamine, counteracts depressive-like behavior induced by corticosterone via PI3K/Akt/mTOR pathway. Mol Neurobiol. (2016) 53:6818–34. 10.1007/s12035-015-9580-9 26660117

[96] Cunha MP Budni J Ludka FK Pazini FL Rosa JM Oliveira A. Involvement of PI3K/Akt signaling pathway and its downstream intracellular targets in the antidepressant-like effect of creatine. Mol Neurobiol. (2016) 53:2954–68. 10.1007/s12035-015-9192-4 25943184

[97] Ludka FK Constantino LC Dal-Cim T Binder LB Zomkowski A Rodrigues AL. Involvement of PI3K/Akt/GSK-3beta and mTOR in the antidepressant-like effect of atorvastatin in mice. J Psychiatr Res. (2016) 82:50–7. 10.1016/j.jpsychires.2016.07.004 27468164

[98] Lima IVA Almeida-Santos AF Ferreira-Vieira TH Aguiar DC Ribeiro FM Campos AC. Antidepressant-like effect of valproic acid-Possible involvement of PI3K/Akt/mTOR pathway. Behav Brain Res. (2017) 329:166–71. 10.1016/j.bbr.2017.04.015 28408298

[99] Kuang WH Dong ZQ Tian LT Li J. IGF-1 defends against chronic-stress induced depression in rat models of chronic unpredictable mild stress through the PI3K/Akt/FoxO3a pathway. Kaohsiung J Med Sci. (2018) 34:370–6. 10.1016/j.kjms.2018.02.004 30063009 PMC11915624

[100] Hu X Tao C Gan Q Zheng J Li H You C. Oxidative stress in intracerebral hemorrhage: sources, mechanisms, and therapeutic targets. Oxid Med Cell Longev. (2016) 2016:3215391. 10.1155/2016/3215391 26843907 PMC4710930

[101] Felger JC Alagbe O Pace TW Woolwine BJ Hu F Raison CL. Early activation of p38 mitogen activated protein kinase is associated with interferon-alpha-induced depression and fatigue. Brain Behav Immun. (2011) 25:1094–8. 10.1016/j.bbi.2011.02.015 21356304 PMC3116018

[102] Cao X Rui L Pennington PR Chlan-Fourney J Jiang Z Wei Z. Serine 209 resides within a putative p38(MAPK) consensus motif and regulates monoamine oxidase-A activity. J Neurochem. (2009) 111:101–10. 10.1111/j.1471-4159.2009.06300.x 19650872

[103] Bruchas MR Schindler AG Shankar H Messinger DI Miyatake M Land BB. Selective p38alpha MAPK deletion in serotonergic neurons produces stress resilience in models of depression and addiction. Neuron (2011) 71:498–511. 10.1016/j.neuron.2011.06.011 21835346 PMC3155685

[104] Jia W Liu R Shi J Wu B Dang W Du Y. Differential regulation of MAPK phosphorylation in the dorsal hippocampus in response to prolonged morphine withdrawal-induced depressive-like symptoms in mice. PLoS ONE (2013) 8:e66111. 10.1371/journal.pone.0066111 23823128 PMC3688859

[105] Park EC Rongo C. The p38 MAP kinase pathway modulates the hypoxia response and glutamate receptor trafficking in aging neurons. Elife (2016) 5:e12010. 10.7554/eLife.12010 26731517 PMC4775213

[106] Martin-Hernandez D Caso JR Bris AG Maus SR Madrigal JL Garcia-Bueno B. Bacterial translocation affects intracellular neuroinflammatory pathways in a depression-like model in rats. Neuropharmacology (2016) 103:122–33. 10.1016/j.neuropharm.2015.12.003 26686392

[107] Reus GZ Vieira FG Abelaira HM Michels M Tomaz DB dos Santos MA. MAPK signaling correlates with the antidepressant effects of ketamine. J Psychiatr Res. (2014) 55:15–21. 10.1016/j.jpsychires.2014.04.010 24819632

[108] Yang JM Rui BB Chen C Chen H Xu TJ Xu WP. Acetylsalicylic acid enhances the anti-inflammatory effect of fluoxetine through inhibition of NF-kappaB, p38-MAPK and ERK1/2 activation in lipopolysaccharide-induced BV-2 microglia cells. Neuroscience (2014) 275:296–304. 10.1016/j.neuroscience.2014.06.016 24952332

[109] Moretti M Budni J Ribeiro CM Rieger DK Leal RB Rodrigues ALS. Subchronic administration of ascorbic acid elicits antidepressant-like effect and modulates cell survival signaling pathways in mice. J Nutr Biochem. (2016) 38:50–6. 10.1016/j.jnutbio.2016.09.004 27721116

[110] Klionsky DJ Emr SD. Autophagy as a regulated pathway of cellular degradation. Science (2000) 290:1717–21. 10.1126/science.290.5497.1717 11099404 PMC2732363

[111] De Duve C Pressman BC Gianetto R Wattiaux R Appelmans F. Tissue fractionation studies. 6. Intracellular distribution patterns of enzymes in rat-liver tissue. Biochem J. (1955) 60:604–17. 13249955 10.1042/bj0600604 PMC1216159

[112] Wang JP Zhang MY. Role for target of rapamycin (mTOR) signal pathway in regulating neuronal injury after intracerebral hemorrhage. Cell Physiol Biochem. (2017) 41:145–53. 10.1159/000455983 28214828

[113] Yu A Zhang T Zhong W Duan H Wang S Ye P. miRNA-144 induces microglial autophagy and inflammation following intracerebral hemorrhage. Immunol Lett. (2017) 182:18–23. 10.1016/j.imlet.2017.01.002 28062218

[114] Wang Z Yuan B Fu F Huang S Yang Z. Hemoglobin enhances miRNA-144 expression and autophagic activation mediated inflammation of microglia via mTOR pathway. Sci Rep. (2017) 7:11861. 10.1038/s41598-017-12067-2 28928406 PMC5605685

[115] Shi H Wang J Wang J Huang Z Yang Z. IL-17A induces autophagy and promotes microglial neuroinflammation through ATG5 and ATG7 in intracerebral hemorrhage. J Neuroimmunol. (2018) 323:143–51. 10.1016/j.jneuroim.2017.07.015 28778418

[116] Abelaira HM Reus GZ Neotti MV Quevedo J. The role of mTOR in depression and antidepressant responses. Life Sci. (2014) 101:10–4. 10.1016/j.lfs.2014.02.014 24582593

[117] Feng P Huang C. Phospholipase D-mTOR signaling is compromised in a rat model of depression. J Psychiatr Res. (2013) 47:579–85. 10.1016/j.jpsychires.2013.01.006 23421961

[118] Wang Q Zhao G Yang Z Liu X Xie P. Downregulation of microRNA1243p suppresses the mTOR signaling pathway by targeting DDIT4 in males with major depressive disorder. Int J Mol Med. (2018) 41:493–500. 10.3892/ijmm.2017.3235 29115444

[119] Yu JJ Zhang Y Wang Y Wen ZY Liu XH Qin J. Inhibition of calcineurin in the prefrontal cortex induced depressive-like behavior through mTOR signaling pathway. Psychopharmacology (2013) 225:361–72. 10.1007/s00213-012-2823-9 22875481

[120] Cui W Ning Y Hong W Wang J Liu Z Li MD. Crosstalk between inflammation and glutamate system in depression: signaling pathway and molecular biomarkers for ketamine's antidepressant effect. Mol Neurobiol. (2018). 10.1007/s12035-018-1306-3. [Epub ahead of print]. 30140973

[121] liu S Li T Liu H Wang X Bo S Xie Y. Resveratrol exerts antidepressant properties in the chronic unpredictable mild stress model through the regulation of oxidative stress and mTOR pathway in the rat hippocampus and prefrontal cortex. Behav Brain Res. (2016) 302:191–9. 10.1016/j.bbr.2016.01.037 26801825

[122] Zhang B Guo F Ma Y Song Y Lin R Shen FY. Activation of D1R/PKA/mTOR signaling cascade in medial prefrontal cortex underlying the antidepressant effects of l-SPD. Sci Rep. (2017) 7:3809. 10.1038/s41598-017-03680-2 28630404 PMC5476681

[123] Shen X Ma L Dong W Wu Q Gao Y Luo C. Autophagy regulates intracerebral hemorrhage induced neural damage via apoptosis and NF-kappaB pathway. Neurochem Int. (2016) 96:100–12. 10.1016/j.neuint.2016.03.004 26964766

[124] Qureshi AI Suri MF Ostrow PT Kim SH Ali Z Shatla AA. Apoptosis as a form of cell death in intracerebral hemorrhage. Neurosurgery (2003) 52:1041–7. 10.1227/01.NEU.0000057694.96978.BC 12699545

[125] Wang YX Yan A Ma ZH Wang Z Zhang B Ping JL. Nuclear factor-kappaB and apoptosis in patients with intracerebral hemorrhage. J Clin Neurosci. (2011) 18:1392–5. 10.1016/j.jocn.2010.11.039 21782444

[126] Matsushita K Meng W Wang X Asahi M Asahi K Moskowitz MA. Evidence for apoptosis after intercerebral hemorrhage in rat striatum. J Cereb Blood Flow Metab. (2000) 20:396–404. 10.1097/00004647-200002000-00022 10698078

[127] Sun H Li L Zhou F Zhu L Ke K Tan X. The member of high temperature requirement family HtrA2 participates in neuronal apoptosis after intracerebral hemorrhage in adult rats. J Mol Histol. (2013) 44:369–79. 10.1007/s10735-013-9489-4 23413020

[128] Wu Z Zou X Zhu W Mao Y Chen L Zhao F. Minocycline is effective in intracerebral hemorrhage by inhibition of apoptosis and autophagy. J Neurol Sci. (2016) 371:88–95. 10.1016/j.jns.2016.10.025 27871457

[129] Ding L Zhang C Masood A Li J Sun J Nadeem A. Protective effects of phosphodiesterase 2 inhibitor on depression- and anxiety-like behaviors: involvement of antioxidant and anti-apoptotic mechanisms. Behav Brain Res. (2014) 268:150–8. 10.1016/j.bbr.2014.03.042 24694839 PMC4075066

[130] Jiang Y Li Z Liu Y Liu X Chang Q Liao Y. Neuroprotective effect of water extract of Panax ginseng on corticosterone-induced apoptosis in PC12 cells and its underlying molecule mechanisms. J Ethnopharmacol. (2015) 159:102–12. 10.1016/j.jep.2014.10.062 25446601

[131] Garabadu D Ahmad A Krishnamurthy S. Risperidone attenuates modified stress-re-stress paradigm-induced mitochondrial dysfunction and apoptosis in rats exhibiting post-traumatic stress disorder-like symptoms. J Mol Neurosci. (2015) 56:299–312. 10.1007/s12031-015-0532-7 25750029

[132] Yucel A Yucel N Ozkanlar S Polat E Kara A Ozcan H. Effect of agomelatine on adult hippocampus apoptosis and neurogenesis using the stress model of rats. Acta Histochem. (2016) 118:299–304. 10.1016/j.acthis.2016.02.007 26970810

[133] Li X Wu T Yu Z Li T Zhang J Zhang Z. Apocynum venetum leaf extract reverses depressive-like behaviors in chronically stressed rats by inhibiting oxidative stress and apoptosis. Biomed Pharmacother. (2018) 100:394–406. 10.1016/j.biopha.2018.01.137 29454288

[134] Demirdas A Naziroglu M Ovey IS Duloxetine reduces oxidative stress, apoptosis, and Ca(2+) entry through modulation of TRPM2 and TRPV1 channels in the hippocampus and dorsal root ganglion of rats. Mol Neurobiol. (2017) 54:4683–95. 10.1007/s12035-016-9992-1 27443158

[135] Zhou L Liu C Wang Z Shen H Wen Z Chen D. Pannexin-1 is involved in neuronal apoptosis and degeneration in experimental intracerebral hemorrhage in rats. Mol Med Rep. (2018) 17:5684–91. 10.3892/mmr.2018.8624 29484398 PMC5866010

[136] Ni M He JG Zhou HY Lu XJ Hu YL Mao L. Pannexin-1 channel dysfunction in the medial prefrontal cortex mediates depressive-like behaviors induced by chronic social defeat stress and administration of mefloquine in mice. Neuropharmacology (2018) 137:256–67. 10.1016/j.neuropharm.2017.12.004 29221793

[137] Shen J Chen X Li H Wang Y Huo K Ke K. p75 neurotrophin receptor and its novel interaction partner, NIX, are involved in neuronal apoptosis after intracerebral hemorrhage. Cell Tissue Res. (2017) 368:13–27. 10.1007/s00441-016-2510-y 27726026

[138] Fujii T Yamamoto N Hori H Hattori K Sasayama D Teraishi T. Support for association between the Ser205Leu polymorphism of p75(NTR) and major depressive disorder. J Hum Genet. (2011) 56:806–9. 10.1038/jhg.2011.107 21938001

[139] Zhou L Deng L Chang NB Dou L Yang CX. Cell apoptosis and proliferation in rat brains after intracerebral hemorrhage: role of Wnt/beta-catenin signaling pathway. Turk J Med Sci. (2014) 44:920–7. 10.3906/sag-1308-100 25552142

[140] Villa RF Ferrari F Moretti A. Post-stroke depression: mechanisms and pharmacological treatment. Pharmacol Ther. (2018) 184:131–44. 10.1016/j.pharmthera.2017.11.005 29128343

[141] Li N Worthmann H Deb M Chen S Weissenborn K. Nitric oxide (NO) and asymmetric dimethylarginine (ADMA): their pathophysiological role and involvement in intracerebral hemorrhage. Neurol Res. (2011) 33:541–8. 10.1179/016164111X13007856084403 21669125

[142] Zhao X Zhang Y Strong R Zhang J Grotta JC Aronowski J. Distinct patterns of intracerebral hemorrhage-induced alterations in NF-kappaB subunit, iNOS, and COX-2 expression. J Neurochem. (2007) 101:652–63. 10.1111/j.1471-4159.2006.04414.x 17250675

[143] Ryu J Pyo H Jou I Joe E. Thrombin induces NO release from cultured rat microglia via protein kinase C, mitogen-activated protein kinase, and NF-kappa B. J Biol Chem. (2000) 275:29955–9. 10.1074/jbc.M001220200 10893407

[144] Lu H Shen J Song X Ge J Cai R Dai A. Protective effect of Pyrroloquinoline Quinone (PQQ) in rat model of intracerebral hemorrhage. Cell Mol Neurobiol. (2015) 35:921–30. 10.1007/s10571-015-0187-5 25820784 PMC11486243

[145] Chang CF Cho S Wang J. (-)-Epicatechin protects hemorrhagic brain via synergistic Nrf2 pathways. Ann Clin Transl Neurol. (2014) 1:258–271. 10.1002/acn3.54 24741667 PMC3984761

[146] Benvenisti-Zarom L Chen-Roetling J Regan RF. Inhibition of the ERK/MAP kinase pathway attenuates heme oxygenase-1 expression and heme-mediated neuronal injury. Neurosci Lett. (2006) 398:230–4. 10.1016/j.neulet.2006.01.003 16443326

[147] Pandey GN Rizavi HS Bhaumik R Ren X. Innate immunity in the postmortem brain of depressed and suicide subjects: role of Toll-like receptors. Brain Behav Immun. (2018). 10.1016/j.bbi.2018.09.024. [Epub ahead of print]. 30266463 PMC6476429

